# Opinion Article: NK Cells in Cutaneous Leishmaniasis: Protection or Damage?

**DOI:** 10.3389/fimmu.2022.933490

**Published:** 2022-07-01

**Authors:** Marton Kaique de Andrade Cavalcante, Rafael de Freitas e Silva, Valéria Rêgo Alves Pereira, Maria Carolina Accioly Brelaz-de-Castro

**Affiliations:** ^1^ Department of Immunology, Aggeu Magalhães Institute - Oswaldo Cruz Foundation, Recife, Brazil; ^2^ Parasitology Laboratory, Federal University of Pernambuco, Vitoria de Santo Antão, Brazil; ^3^ Department of Natural Sciences, University of Pernambuco, Garanhuns, Brazil

**Keywords:** natural killer, cutaneous leishmaniasis, immune response, tegumentary leishmaniasis, leishmania

## Introduction: CL and the Early Immune Response

Cutaneous Leishmaniasis (CL) is considered a neglected disease, mainly linked to low socioeconomic conditions (1, 2). Currently, estimates suggest 900,000 to 1.5 million new cases per year with 95% of the cases in the Americas, Mediterranean and Asia (3).

The disease clinical manifestation can range from a single to multiple lesions, being observed in 90% of cases a nodular ulcerative squamous lesion. After inoculation of the metacyclic promastigote of *Leishmania* into the host skin by the sandfly bite, a papule forms at the site of bite, and later it turns into an ulcerated lesion with delineated borders, a reddish background, and an intense inflammatory infiltration (lymphocytes, phagocytes, and plasma cells) (4–9).. Factors such as the species of *Leishmania* involved in the infection and the host immune response directly influence the lesion type and clinical outcome in the patient (10, 11).

After dermis inoculation the promastigotes cause activation of the complement system and factor C3b deposition on the parasite surface. However it has been demonstrated that *Leishmania* protease GP63 is able to inactivate C3b (12). These promastigotes are opsonized, by the C3 molecule of the complement system, to be phagocytized by phagocytic immune cells. Among these phagocytic cells are neutrophils, dendritic cells, and macrophages (13–15).

Neutrophils are the first cells to arrive at the infection site, being attracted by complement proteins, cytokines (e.g., IL-8), and chemokines (e.g., CXCL1 and CXCL2). These cells can eliminate the parasites through the action of nitric oxide (NO) and other reactive oxygen species (ROS), and also produce high levels of chemokines such as CXCL8 and CXCL9, responsible for the recruitment of more neutrophils and Th1 cells (16–18). In addition, because they are short-lived phagocytic cells, they promote the entry of more promastigotes in macrophages phagocyting dead neutrophils. Thus, neutrophils serve as a “trojan horses”, but the macrophages also produce cytokines which will in turn activate other immune cells (19, 20).

Resident dendritic cells (DCs) seems to play a key role in the immunopathogenesis of CL (21, 22). Once these cells interact with *Leishmania* there is an increase in the expression of co-stimulatory molecules, such as CD40, CD80 and CD86 which are essential for T cell activation (23, 24). Thus, it is hypothesized that resident DCs recruit monocytes, which differentiate into monocyte-derived dendritic cells (moDCs). These cells have an intense phagocytic activity, and evidences suggests that they favor the growth and survival of parasites (25, 26).

When *Leishmania* spp. are phagocytized, they are trapped in vesicles called phagosomes, which then fuse with lysosomes and turn into acidic phagolysosomes. From this point on, cellular processes such as oxidative stress and nitric oxide (NO) production take place, thus making the phagolysosome very acidic and hydrolytic. Promastigotes, which are sensitive to acidic and hydrolytic environments, begin to be eliminated. However, as a survival strategy, the promastigotes begin to transform into the form of amastigotes, since the latter are more resistant (13, 27).

The infected macrophages stimulate the production of pro-inflammatory cytokines (IL-1, TNF, IL-18 and IL-12) and chemokines (CXCL10, CCL4, CCL8, CCL11 and CXCL8), which also act to kill the parasite. At the same time the adaptive immune response induce activation of T cells which produce a cellular immune response, and the B-lymphocytes will be involved in the production of antibodies (28, 29).

## Natural Killer Cells

The NK cells are innate immune cells which correspond to 5-20% of circulating lymphocytes in the blood (30–32). These cells can be found in various locations in the body such as liver, bone marrow, and thymus (33) and can directly kill infected or modified tumor cells, in addition to their cytokine-producing function (30, 34).

NK cells are phenotypically known to express CD56 and CD16 and lack CD3 expression in humans. In mouse they express NK1.1 (NKR-P1C), NCR1 (NKp45/CD335) and CD49b, and there is no expression of CD56 by rodent NK cells (35–38). Natural Killer T cells (NKT) are a less frequent subpopulation (0.1-0.5% of peripheral blood leukocytes) of NKs characterized by CD3 and CD56 expression (38–41). NKT cells are T lymphocytes that possess characteristics of both these cells and NK cells, thus adding function of both cell types. There are two types of NKT cells: type I NKT or invariant NKT (iNKT), which express and invariant TCR with the Vα24-Jα18 segment associated with the Vβ11 chain, and type II NKT or non-invariant NKT cells, which express the TCR with varying TCRs on their cell surface (42–44). Among the characteristics of an NKT cell are the presence of the invariant TCR, expression of CD1d, and high production of cytokines, especially IFN-γ, TNF, IL-4, IL-10, and IL-13 (45–49).

## Why Does the Study of NK and NKT Cells Are Important in Cutaneous Leishmaniasis?

**Figure 1:**
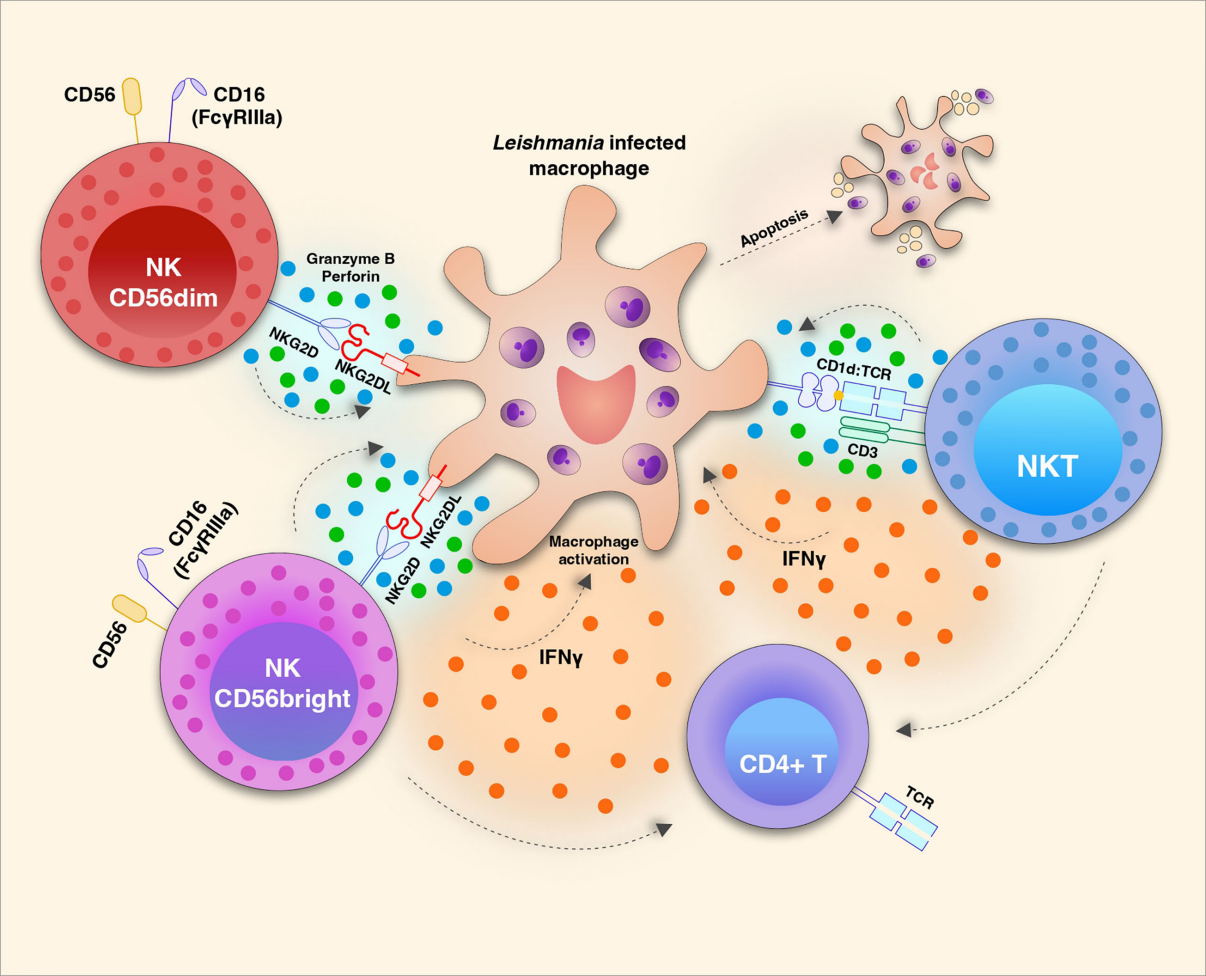
Natural killer (NK) cell subsets play distinct roles during Leishmania infection. NKG2D/NKG2D ligand (L) interaction triggers the effector function of CD56^dim^ (also CD16^+^ in humans) NK cells to release cytotoxic granules with Granzyme b and Perforin which induces apoptosis of *Leishmania* infected macrophages. It is not yet clear tough if this mechanism restricts parasite growth or amplify amastigote proliferation. The same receptor-ligand interaction induces the activation of CD56^bright^ NK cells which are strong producers of cytokines such as IFN-γ and TNF which in turn can act in the activation of macrophages and T cells. The potential recognition of glycolipid antigens present by CD1d to the invariant TCR of NKT cells may induce the production and release of IFN-γ and also apoptotic factors Granzyme B and Perforin.

NK cells are part of the innate immune response and are known for their ability to kill infected cells, as well as to produce cytokines that act in the activation of other immune cells (**Figure 1**). For some time, studies have been trying to dissect the role of NK cells in CL, as well as their contributions to the cure or progression of the disease.

Some authors suggest that these cells act to fight infection due to their cytotoxic role, however, other authors suggest that these cells are also responsible for more collateral damage contributing to lesion exacerbation (50). To tackle this problem, a 5-year cohort study observed that even after clinical cure of CL, the patients’ NK cells continue to produce Interferon-γ (IFN-γ), and this was associated with protection against CL (51). IFN-γ is key-cytokine for macrophage activation and consequent parasite clearance (52).

It has also been shown that the *in vitro* stimulation of peripheral blood mononuclear cells from healthy individuals with *Leishmania* antigen/peptides induces an increase in the frequency of NK cells specially (53, 54). On the other hand, Lieke and colleagues observed a decrease in NK cell proliferation in animal and human models using *L. major* and *L. aethiopica* where the promastigote form of *Leishmania* inhibited proliferation of isolated naive NK cells (55, 56).

NK cells are strong producers of cytokines and cytotoxic granules such as granzyme and perforin and surface markers such as NKG2D, triggering activation of other cells, increasing phagocytosis and parasite elimination (57). However, paradoxically, some works have shown evidence that these NK-derived effector molecules play a key role in the immunopathogenesis of CL, in addition to the inflammation developed. It was observed that patients with active lesion present a higher frequency of these cells, which in turn is directly associated with high levels of IFN-γ, Tumor Necrosis Factor (TNF), as well as granzyme A, granzyme B, granulysin and perforin. For this reason, it was found that most of the cytotoxic activity generated in CL is related to NK and not only to CD8^+^ T cells (58–60). Another study showed that there is no change in the frequency of NK cells according to the period of infection, but in patients who are on treatment as well as in clinically cured patients there is a reduction in their degranulation potential (61).

There seems to be a divergent role of NK cells according to the clinical form that the patient displays. It has been seen that patients with the diffuse form of the disease have a lower frequency of NK cells, also exhibiting lower levels of cytokines such as IFN-γ and TLR expression such as TLR1, TLR2 and TLR6 (62, 63). This fact seems to be related to the severity of the disease. Unlike patients who present the localized form, who exhibit high levels of cytokines, besides a higher frequency of NK cells (63).

Experimental models have demonstrated that depleting NK cells in C57BL/6 mice through the administration of anti-asialo-GM1 or NK1.1 antibodies induces lesion exacerbation in the first weeks of infection. In addition, these animals showed swelling in the local tissue and higher parasite numbers when compared to normal animals (64). NK cell depletion has also been shown to lead to a reduction in IFN-γ levels, which may compromise Th1 response development (64, 65).

Studies on the role of NKT in *Leishmania* infection are still scarce (48). An *in vitro* study used monocyte-derived DCs generated from healthy donors’ buffy coats observed that *Leishmania infantum* infected DCs increase the expression of CD1d, causing NKT cells to recognize these cells. In addition, the researchers observed that the percentage of NKT cells producing IFN-γ was twice as high as that of IL-4-producing cells. Thus, it can be seen that NKT cells can act both in the production of cytokines for the activation of other immune cells, such as T lymphocytes, as well as act in a cytotoxic way on infected cells that cannot be lysed by conventional NK cells (66).

Studies have observed subpopulations of NKT cells that express CD4 and CD8 markers, where these subpopulations have distinct functions. While CD4+ NKT cells are strong and potent producers of cytokines such as IL-2, IL-4 and IL-13, CD8^+^ NKT cells act more aggressively in fighting infection, through their cytotoxic activity (67–69). Studies by Gumperz et al. (67) and Carvalho et al. (70) observed higher numbers of CD8+ NKT cells in healthy individuals (67, 70).

Recently it has been shown that antigen from *Leishmania braziliensis* is able to induce activation of NKT cells in peripheral blood mononuclear cells (PBMC) from patients with CL in addition to CD107a+ NKT cells, thus suggesting that these cells may be involved in inflammation and consequently lesion formation (59). This is confirmed by Ferraz and colleagues (2017) who observed the presence of these cells at the site of injury (71). CD107a is a surface marker found on NK cells as well as CD8+ T cells and is used to assess the degranulation of these cells, allowing one to investigate whether or not there is granule release and consequent lysis of infected cells (72, 73).

Currently, there is no consensus on the true function of NK and NKT cells in CL. The data presented by the studies suggest that NK cells appear to contribute strongly to a toxic environment and consequently to the development of lesions. At the same time, these cells seem to be crucial for the development of a cellular immune response, in view of their high production of IFN-γ, which helps in the activation of cells of the adaptive immune response. Studies to understand the factors that lead NK cells to generate a balance at the site of injury are strongly recommended.

Thus, more comprehensive research on these cells and their interaction with *Leishmania* is urgent. For example, single-cell RNA sequencing studies are necessary and can help understand the contribution of NK cells to lesion formation or resolution in CL. Moreover, those studies would foster the development of new treatments, diagnostics and vaccines to effectively combat CL.

## Author Contributions

MC, RS and MB-D-C wrote the manuscript. VP contributed to the discussion of the draft and made final corrections. All authors contributed to the article and approved the submitted version.

## Funding

This work was supported by UFPE-Propesqi nº 12/2021. MC is the recipient of a Ph.D. fellowship from FACEPE.

## Conflict of Interest

The authors declare that the research was conducted in the absence of any commercial or financial relationships that could be construed as a potential conflict of interest.

## Publisher’s Note

All claims expressed in this article are solely those of the authors and do not necessarily represent those of their affiliated organizations, or those of the publisher, the editors and the reviewers. Any product that may be evaluated in this article, or claim that may be made by its manufacturer, is not guaranteed or endorsed by the publisher.
